# Invasive procedures and atraumatic care in pediatric nursing practice: nurses’ perceptions

**DOI:** 10.3389/fped.2025.1543138

**Published:** 2025-05-15

**Authors:** Júlia Neto, Rita Fernandes, Luísa Andrade, Ilda Fernandes, Teresa Martins, Maria do Céu Barbieri-Figueiredo, Fernanda Carvalho, Lígia Lima

**Affiliations:** ^1^ Escola Superior de Enfermagem do Porto, RISE-Health, Porto, Portugal; ^2^ Departamento de Enfermería, Universidade de Huelva, Huelva, Spain

**Keywords:** invasive procedures, stress, pain, anxiety, child, nursing, non-pharmacological strategies

## Abstract

**Introduction:**

Invasive procedures in pediatric nursing practice require a child-centered approach to minimize pain levels associated with manifestations of stress, anxiety, and long-term traumatic reactions.

**Method:**

This cross-sectional study aimed at identifying nurses’ perception of stress, anxiety, and pain levels in children and adolescents (0–18 years) undergoing invasive procedures, and strategies used to minimize the associated distress. Based on a literature review, an online questionnaire was developed that evaluates the distress associated with frequent invasive procedures and strategies used to reduce discomfort.

**Results:**

Participants were 157 nurses who provided nursing care to children in all types of healthcare settings, such as hospitals and community health centers. Nurses evaluated lumbar puncture and catheter insertion as the procedures that caused more stress, anxiety, and pain in all age groups. ANOVA and *post-hoc* analyses indicate that nurses perceived adolescents as experiencing less stress than children in all the invasive procedures. Pediatric specialist nurses perceived a significantly higher total level of distress (an index that indicates stress, anxiety or pain in the whole group of procedures) compared to nurses with other specialties or generalist nurses, in all age groups. Non-pharmacological strategies were the most frequently used strategies used by nurses for minimizing distress in children, including distraction (51.2%), preparation (30.7%), and sensory techniques (14.6%). Pharmacological strategies, such as topical anesthetics and light sedation, were less frequently used.

**Conclusion:**

This study highlights the importance of specialized training and knowledge concerning strategies to reduce distress in pediatric invasive procedures, suggesting the need for more significant investment in education and support for nursing professionals to improve patient experience.

## Introduction

Invasive procedures in the pediatric context represent a challenge in healthcare, requiring a child-centered approach to minimizing the associated physical and emotional impacts. Invasive procedures are all those that involve needles, the insertion of probes into natural orifices or that cause actual or potential tissue damage, except major surgical and dental procedures (1) carried out in the context of primary health care or hospital care. In most clinical situations, these procedures are related to interventions for the diagnosis and treatment of health conditions and illnesses, such as the insertion of catheters, blood sampling, and lumbar punctures, among others (1).

The invasive nature of the procedures means that the child experiences an unpleasant sensory and emotional experience resulting from actual or potential tissue damage (1), causing physical and/or emotional impact. Differentiated levels of pain are associated with manifestations of stress, fear, anxiety, and long-term traumatic reactions (2, 3) and are related to age, gender, previous experiences, culture, type of illness, and family characteristics, among others (4–7). When dealing with invasive procedures, nursing interventions should focus on the atraumatic care paradigm, to minimize or eliminate physical and/or emotional impact. It is essential to reduce the perception of pain as much as possible, respecting the children's cognitive and emotional development, and promoting their safety and comfort (1, 4, 8, 9). This can be mediated by strategies promoting the humanization of care based on the following principles: preventing or minimizing the separation of the child from the family; promoting his/her involvement and ensuring his/her participation, ensuring the partnership of care or family-centered care, and minimizing the impact of bodily injury and pain (2, 10, 11).

Scientific evidence has shown that the use of these strategies results in lower levels of anxiety and discomfort in children and better cooperation during procedures (1, 4, 12), which underpins the need for their selection to be based on a careful assessment of the human resources, namely the training and experience of nursing staff, and available materials (1). Nurses use pharmacological and non-pharmacological strategies, in isolation or combined, to minimize pain, stress, and fear of invasive procedures. Pharmacological strategies are used depending on the degree of invasiveness and duration of the procedure, the expected pain, the child's level of anxiety, the degree of immobility required, and whether the procedure is expected to be repeated (1), examples being topical analgesia (EMLA®), local infiltration (1%–2% buffered Lidocaine) and systemic analgesia (1). Non-pharmacological strategies ensure that the child comprehends the procedure through cognitive restructuring, aimed at the cognitions, expectations, evaluations, and constructions that accompany the experience of pain, modifying the cognitions responsible for reactions of fear, anxiety and depression (4). Depending on the modulation of the painful stimulus, the approach of non-pharmacologic strategies is structured as follows: (i) behavioral: relaxation, cognitive, anticipatory information; (ii) cognitive-behavioral: distraction and guided imagination, such as non-nutritive suction, breastfeeding, administration of 30% glucose or 24% sucrose, use of therapeutic toys; (iii) physical or peripheral: superficial dry or moist heat, cold, superficial touch/massage, positioning or transcutaneous electrical nerve stimulation; (iv) emotional support: the presence of a family member or significant person, positive reinforcement; and (v) environmental: light, noise, temperature and decoration (1, 4, 13, 14).

Nurses must gain knowledge on how to recognize procedures that generate stress, anxiety or pain, based on the children's singularities, considering their best interests and improving their well-being about invasive procedures. Therefore, nurses caring for children should act in compliance with the Charter for Children in Hospitals, avoiding unnecessary examinations or treatments; and minimizing physical and emotional aggression, about pain and its physical and emotional impact (15) and the Quality Standards, referring to the descriptive statement “well-being and self-care”, recommended by the Portuguese Order of Nurses (16). A study carried out on how nurses evaluate the use of play and therapeutic toys by the nursing team in sick children care, points out that these non-pharmacological strategies present advances and challenges in terms of the use of dolls, dramatization, and distraction to carry out procedures, the need for colorful and fun uniforms and also recognizes the potential of play and the challenging barriers that nurses face and that emerge from the link between play and nursing care (17). Another study looked at nurses' perceptions of the use of therapeutic toys in children with fully implanted central venous catheters, whose insertion and puncture generate anxiety, stress, and other reactions. The main conclusions report the importance of adapting the material used to make the toy, making it easy to wash/disinfect to minimize cross-infections; adapting the toy to take into account the diversity of gender, race, age, and catheter insertion site; and the need for training/dissemination of knowledge to empower professionals about the impact and quality of care provided to children (18).

This research aimed at: (i) describing the invasive procedures that, from the nurses' perspective, generate the most stress, anxiety, or pain in different pediatric age groups; (ii) identifying the strategies used to promote comfort during these procedures; and analyzing possible differences in the nurses' perception of the impact of invasive procedures, considering sociodemographic variables such as age, length of service and specific training in child and pediatric health.

## Material and methods

### Study design

This is a cross-sectional, correlational study.

### Participants

Participants of this study were Portuguese nurses who provided nursing care to children in all types of healthcare settings, such as hospitals and community health centers. This cross-sectional study used convenience sampling to disseminate the survey hyperlink across multiple online channels, using snowball technique, including WhatsApp, Facebook, and personal emails. The study covered diverse geographical areas across Portugal. The data collection phase extended from February to April 2024.

### Instrument

The research team developed the questionnaire based on a literature review specifically for this study. It included questions related to the participants' sociodemographic variables, specifically age, gender, length of professional experience in pediatrics/child health, current professional context (hospital or primary health care), training in child health/pediatrics, and pain.

To assess the nurses' perception of the level of distress (stress, anxiety, and pain) associated with invasive procedures, based on the Portuguese Directorate-General of Health technical guidelines (1) on pain control in invasive procedures in children, twelve procedures were selected, more specifically: treatment of simple wounds; treatment of complex wounds; removal of drains or sutures; aspiration of secretions; a collection of secretions with a swab; peripheral venipuncture (pvp); capillary puncture; lumbar puncture; removal of dressings and adhesives; vaccination/intramuscular injection/subcutaneous injection; placement of a peripherally inserted central catheter (epicutaneous); ureteral/oro-naso-gastric catheterization. Participants were asked to rate each of the procedures on a Likert scale with five response options (from none to extreme distress) considering the following age groups: 0-to 1-year; 1-to-5-years; 6-to-10-years; and 11-to-18 years. For each age group, an option (open-ended) was also introduced, in which participants could describe procedures other than those included in the questionnaire that, in their opinion, also caused stress, anxiety, or pain in children/adolescents. Finally, the questionnaire also included an open-ended question in which the participants were asked to describe the measures they used in their clinical practice to minimize distress (stress, anxiety, and pain) when performing invasive procedures.

### Data analysis

Statistical analyses were performed using SPSS for Windows, Version 28.0. To determine the sample size, we used G*Power 3.1.9.7 with the intention of conducting an ANOVA, assuming a medium effect size of 0.25, an alpha error of 0.005, and a power (1-*β*) of 0.80. The determined sample size was 159 participants. Categorical variables were presented as percentages, while continuous variables were reported as means (M) and standard deviations (SD). To assess group differences, ANOVA was conducted, followed by *post hoc* Bonferroni tests. A *p*-value of 0.05 was considered the threshold for statistical significance. The internal consistency of the questionnaire items related to the assessment of stress associated with 12 invasive procedures was calculated using Cronbach's alpha coefficient. A qualitative analysis was performed to analyze the responses to the open-ended question regarding the strategies used by nurses to minimize childreńs distress when experiencing invasive procedures.

### Ethical issues

The study followed the Declaration of Helsinki statements. All the ethical considerations associated with the study of human beings were respected, including the permission from the Ethics Committee of the authors' host institution (Flow CE_32/2023). Participation was voluntary, and nurses were informed about the study's aims and assured of the anonymity of their input.

## Results

### Sociodemographic characteristics of the participants

Questionnaires with more than 10% missing responses were excluded, resulting in a final sample of 157 participants. The study participants (Table 1) were predominantly female (94%), with a mean of 42.9 years (±10.1). Most of them worked in hospital settings (68%), while 27% were employed in Primary Health Care units. Geographically, the majority were from the North region (50%), followed by Lisbon and the Tagus Valley (16%), and the Centre region (15%). Participants' experience in child health and pediatrics ranged from less than one year to 40 years, with an average of 16.6 years (±9.9). Regarding their educational background, 42% held a bachelor's degree, and 52% had a master's and/or nursing specialization in child health and pediatrics. Additionally, the majority (70%) had training in pediatric pain management.

**Table 1:** Sociodemographic characteristics of the participants.

Participants	Range	M(SD)
Age	Min = 23 e Max = 67	42.9 (10.1)
Experience in pediatrics/pediatric nursing (years)	Min < 1 e Max. = 40 years	16.6 (9.9)
	N	%
Sex		
Female	148	94
Male	9	6
Professional Setting		
Hospital (in-patient, outpatient, emergency, ..)	108	68.8
Primary Health Care	42	26.8
Other	7	4.5
Geographic location		
North	79	50.3
Centre	24	15.3
Lisbon and Tagus Valey	25	15.9
Alentejo	2	1,3
Algarve	4	2.
Madeira Autonomous Region	15	9.6
Azores Autonomous Region	8	15.9
Nursing Education		
BSC	41	26,1
MSc Pediatric Nursing/Specialization in Pediatric Nursing	80	51
Specialization in another clinical area	36	22,9
Training in pediatric pain management		
Yes	110	70
No	47	30

### The invasive procedures that, from the nurses' perspective, trigger the most stress, anxiety, or pain, in different age groups

The internal consistency of the questionnaire items assessing stress related to 12 invasive procedures was evaluated using Cronbach's alpha coefficient. The internal consistency values ranged from 0.94 for the 0-to-1-year age group to 0.95 for the remaining age groups.

An analysis of the mean score for stress, anxiety, or pain levels associated with the different invasive procedures across the four age groups (Table 2) revealed that in the three youngest age groups, the procedures mentioned as most challenging were the same and in descending order, lumbar puncture, insertion of nasogastric tubes and catheters, aspiration of secretions and peripheral venipuncture. In adolescents, lumbar puncture, the insertion of nasogastric tubes and catheters, and aspiration of secretions had the same order, but peripheral venipuncture (PVP) was considered to involve a lower degree of stress, anxiety, or pain. In all age groups, the treatment of simple wounds and the removal of adhesives were the procedures that, according to the nurseś perceptions, caused the least stress, anxiety, or pain, but in the youngest age group (0-to-1 year), the removal of adhesives had a much higher stress, anxiety or pain score compared to the other groups.

**Table 2:** Analysis of differences in nurses’ perceptions of stress, anxiety, and pain across procedures and age groups using ANOVA and *post hoc* tests.

Invasive procedures	Age groups	Mean	SD	ANOVA F(df); p	*post hoc*
Simple wound care	0-to-1 year^a^	2.94	(0.87)	24.53 (3.576); 0.001	a≠d
	1-to-5 years^b^	3.20	(0.92)		b≠c; b≠d
	6-to-10 years^c^	2.80	(0.93)		c≠b; c≠d
	11-to-18 years^d^	2.32	(0.85)		d≠a; d≠b; d≠c
Complex wound care	0-to-1 year^a^	3.77	(0.99)	18.92 (3.528); 0.001	a≠d
	1-to-5 years^b^	4.00	(0.90)		b≠d
	6-to-10 years^c^	3.71	(0.85)		c≠d
	11-to-18 years^d^	3.18	(0.91)		d≠a; d≠b; d≠c
Removal of drains/sutures	0-to-1 year^a^	3.69	(1.00)	15.01 (3.550); 0.001	a≠d
	1-to-5 years^b^	3.96	(0.93)		b≠d
	6-to-10 years^c^	3.68	(0.92)		c≠d
	11-to-18 years^d^	3.20	(0.96)		d≠a; d≠b; d≠c
Aspiration of secretions/suctioning	0-to-1 year^a^	3.96	(1.00)	5.42 (3.479); 0.001	a≠d
	1-to-5 years^b^	4.07	(0.90)		b≠d
	6-to-10 years^c^	3.90	(0.96)		
	11-to-18 years^d^	3.56	(1.08)		d≠a; d≠b
Collection of secretions with a swab	0-to-1 year^a^	3,.55	(0.98)	17.01 (3.540); 0.001	a≠d
	1-to-5 years^b^	379	(0.90)		b≠d
	6-to-10 years^c^	3.52	(0.88)		c≠d
	11-to-18 years^d^	3.00	(0.96)		d≠a; d≠b; d≠c
Peripheral venipuncture	0-to-1 year^a^	3.96	(0.94)	23.87 (3.522); 0.001	a≠d
	1-to-5 years^b^	4.10	(0.81)		b≠c; b≠d
	6-to-10 years^c^	3.77	(0.86)		c≠b; c≠d
	11-to-18 years^d^	3.25	(0.85)		d≠a; d≠b; d≠c
Capillary puncture	0-to-1 year^a^	3.49	(1.06)	13.38 (3.477); 0.001	a≠d
	1-to-5 years^b^	3.63	(0.98)		b≠d
	6-to-10 years^c^	3.33	(1.02)		c≠d
	11-to-18 years^d^	2.84	(0.97)		d≠a; d≠b; d≠c
Lumbar puncture	0-to-1 year^a^	4.15	(1.07)	3.50 (3.423); 0.016	
	1-to-5 years^b^	4.37	(0.90)		b≠d
	6-to-10 years^c^	4.25	(1.02)		
	11-to-18 years^d^	3.93	(1.01)		d≠b
Removal of dressings and adhesives	0-to-1 year^a^	3.17	(1.03)	20.33 (3.583); 0.001	a≠d
	1-to-5 years^b^	3.27	(1.00)		b≠c; b≠d
	6-to-10 years^c^	2.94	(1.06)		c≠b; c≠d
	11-to-18 years^d^	2.41	(1.05)		d≠a; d≠b; d≠c
Vaccination/intramuscular injection/subcutaneous injection	0-to-1 year^a^	3.67	(0.94)	16.29 (3.570); 0.001	a≠d
	1-to-5 years^b^	3.85	(1.01)		b≠d
	6-to-10 years^c^	3.55	(0.97)		c≠d
	11-to-18 years^d^	3.07	(1.02)		d≠a; d≠b; d≠c
Placement of a peripherally inserted central catheter	0-to-1 year^a^	4.04	(1.09)	3.30 (3.376); 0.02	
	1-to-5 years^b^	4.19	(1.05)		b≠d
	6-to-10 years^c^	4.07	(1.06)		
	11-to-18 years^d^	3.71	(1.08)		d≠b
Ureteral/oro-naso-gastric catheterization	0-to-1 year^a^	3.89	(1.11)	4.16 (3.454); 0.006	
	1-to-5 years^b^	4.23	(0.98)		b≠d
	6-to-10 years^c^	4.14	(1.00)		
	11-to-18 years^d^	3.82	(1.02)		d≠b

An analysis of differences in means (ANOVA) revealed statistically significant differences between the age groups in all the procedures analyzed (Table 2), suggesting that age significantly influences negative reactions to the different procedures. *post hoc* tests (represented by comparisons between groups “a≠d”, “b≠c”, etc.) indicate where these differences occurred. For example, for mobilizing drains and performing sutures, there was a significant difference between the 0-to-1-year group (a) and the 11-to-18-year group (d), as well as between other age groups.

Analyzing the differences between the mean scores for all the procedures (ANOVA and Post-hocs), adolescence was the age group in which nurses perceived the least stress, anxiety and pain, almost always with significant differences compared to the other age groups.

Participants had the option of adding other procedures that caused stress, anxiety, or pain, in addition to those considered in the questionnaire. From this analysis, it was evident that in all age groups, endotracheal intubation and chest or abscess drainage were reported as causing extreme levels of distress (stress, anxiety, or pain). Moreover, in younger children (0-to-1 year, 1-to-5 years) and adolescents, in addition to the procedures mentioned above, skin biopsy and the puncture of a fully implanted central venous catheter were also cited as causing extreme distress.

Other procedures perceived as very disturbing procedures in these age groups were non-invasive ventilation, preparation for invasive ventilation, electrode and oximetry placement, bladder puncture, plaster placement, nasal lavage, and undressing. With a moderate degree of distress, the participants also mentioned enemas and rectal stimulation.

In the 6-to-10-year-old group, oral medication and nasal lavage were considered to be procedures that triggered extreme distress, while observation by an otolaryngologist and immobilization due to fractures were considered to cause a great deal of distress. These results suggest that nurses perceive the stress, anxiety or pain caused by different procedures to be different depending on the age of the children and adolescents, possibly reflecting different patterns of response or adaptation to medical treatments between age groups.

### Differences between nurses' perception of the degree of stress, anxiety, or pain caused by different invasive procedures according to sociodemographic variables

A total index of nurses' perception of the distress of the different invasive procedures was calculated for each age group, adding up the level of distress associated with each invasive procedure analyzed. No statistically significant associations were found between the total level of stress, anxiety, or pain caused by the different procedures and the nurses' age or length of service. Significant differences were observed in the total levels of stress, anxiety, or pain based on nursing education, analyzed across three groups: nurses with a degree, nurses with a specialty in child and pediatric health, and a third group with other specialties. Table 3 shows that, except for the adolescent age group, pediatric specialist nurses perceived a higher total level of distress (stress, anxiety, or pain) in all other age groups compared to nurses with other specialties or generalist nurses.

**Table 3:** Analysis of differences in nurses’ perceptions of overall stress scores across age groups, considering nurses’ training, using ANOVA and *post hoc* tests.

Age group	Nurses’ training	N	Mean	SD	ANOVA F(df); p	*post hocs*
0-to-1 year	Bachelor^a^	41	32.90	(12.50)	8.18 (<0.001)	a≠b
	MSc/Pediatric Nursing Specialist^b^	80	42.06	(11.39)		b≠a; b≠c
	Other specialized training^c^	36	35.06	(15.95)		c≠b
1-to-5 year	Bachelor^a^	41	33.05	(14.25)	3.60 (0.03)	a≠b
	MSc/Pediatric Nursing Specialist^b^	80	40.06	(16.06)		b≠a; b≠c
	Other specialized training^c^	36	33.06	(19.09)		c≠b
6-to-10 year	Bachelor^a^	41	30.29	(14.21)	3.68 (0.027)	a≠b
	MSc/Pediatric Nursing Specialist^b^	80	37.28	(14.55)		b≠a; b≠c
	Other specialized training^c^	36	30.75	(19.17)		c≠b
11-to-18 year	Bachelor^a^	41	27.10	(13.89)	2.09 (0.127)	
	MSc/Pediatric Nursing Specialist^b^	80	31.96	(12.98)		
	Other specialized training^c^	36	27.44	(17.72)		

### Strategies used by nurses to promote comfort during invasive procedures

The qualitative analysis of the answers to the open question: “Identify the measures you use in your clinical practice to reduce distress during invasive procedures”, allowed the identification of two main groups of strategies: pharmacological and non-pharmacological (Table 4). In the non-pharmacological strategies, which represent the vast majority of the strategies reported by the nurses, different subgroups were identified, in decreasing order of frequency: (a) cognitive-behavioral strategies, considering in this group distraction techniques (e.g., with word games or using a device with games), preparation for the procedure (e.g., through a demonstration beforehand or an explanation appropriate to the child's age), relaxation techniques (e.g., through breathing exercises or handling anti-stress toys), positive reinforcement (e.g., giving a reward such as a sticker or a diploma); and empowerment (e.g., allowing choosing where they wanted the procedure to be carried out, for example being punctured); (b) sensory strategies through sucking (e.g., using a pacifier or breastfeeding), touching (e.g., massage or kangaroo technique), listening (e.g., music), and managing the environment (e.g., controlled stimulation, e.g., through lighting). controlled stimulation, e.g., through soft light; and (c) emotional support strategies such as the presence and collaboration of parents (e.g., in positioning the child) and the use of comfort objects (e.g., allowing the child to be in contact with their toy or security blanket).

**Table 4:** Frequency and types of comfort-promoting strategies used by nurses during invasive procedures.

Strategies used by nurses			(*N*)	(%)	% by type of strategies
Non-pharmacologic strategies					
	Cognitive-behaviorals	Distraction	147	51.2	50
		Preparation	88	30.7	
		Relaxation	31	10.8	
		Positive reinforcement	11	3.8	
		Empowerment	10	3.5	
	Sensory	Non-nutritive suction	84	14.6	29.9
		Touching	51	8.9	
		Listening	27	4.7	
		Managing the environment	10	1.7	
	Emotional support	Parents presence	45	7.8	9.7
		Parents collaboration	10	1.7	
		Use of meaningful objects	1	0.2	
Pharmacologic strategies	Drugs	Topical anesthetics	31	5.4	10.1
		Analgesia	13	2.3	
		Light sedation	6	1.0	
		Inhaled sedation/analgesia	5	0.9	
		Skin protectors and adhesive removers	3	0.5	
None strategy	None		2	0.4	0.4
	Total		575	100.0	100.0

Less frequently described, pharmacological strategies included topical anesthetics (cream, dressing, or spray); the use of an inhaled sedative/analgesic such as nitrous protoxide; as well as light sedation and other unspecified drugs, in addition to skin protectors and adhesive removers.

Two participants reported that they did not use any measures to promote comfort during invasive procedures.

## Discussion

### Invasive procedures that generate greater stress, anxiety, or pain

According to the perception of the nurses who took part in this study, invasive procedures performed on children triggered different levels of stress, anxiety, or pain, depending on the type of procedure and the age group of the children/adolescents. Of these, lumbar puncture, the insertion of nasogastric tubes and catheters, aspiration of secretions, and peripheral venipuncture, along with endotracheal intubation, were the most challenging procedures at all ages. Similarly, in a study that characterized the invasive procedures commonly carried out on children in a hospital setting, the procedures that caused the greatest degree of pain (moderate to severe) were endotracheal tube insertion and removal, central and peripheral catheter insertion, lumbar puncture, endoscopy, intramuscular injection, and wound treatment (19). In neonates, nurses considered all the following procedures to be painful, in descending order: insert chest tube, lumbar puncture, Insertion surgically inserted central line, bladder catheterisation, insertion arterial line, endotracheal intubation; arterial blood gas, IV cannula insertion, venipuncture, among others (20).

In the context of decision-making for pain management during invasive procedures in pediatric care, guidelines serve as fundamental directives and are formulated based on the healthcare contexts of various countries, including Portugal (1), Canada (21), and Australia (22).These guidelines share the common goal of ensuring quality, safety, and standardization of care, minimizing variations in clinical practice, and promoting better outcomes in pediatric healthcare.

Pain is a particularly complex phenomenon in children due to specific aspects such as their stage of development and previous experiences, among others (23, 24). In this sense, and considering the stage of development, the results of this study highlighted the greater vulnerability of children between zero and one year of age to the removal of adhesives or treatment of simple wounds compared to other age groups. This more intense reaction may be associated with the fragility of the skin in this age group (25), which emphasizes the importance of specific techniques in apparently simple procedures. In children younger than 3 years old, an essential period for child development, invasive procedures without adequate pain management strategies may cause impacts throughout life (26). In pediatric oncology, children, parents, and nurses perceived pain related to procedures and treatments as a bigger problem than the disease itself. The procedures mentioned were removal of sticking plasters, venipunctures, intramuscular injection, procedures related to internal central lines and nasogastric tubes, postoperative pain, with a more significant impact on children under five, associated with their lower cognitive maturity and difficulty in dealing with fear and pain (27). Fear of needles is the most common fear in the pediatric population, particularly in the school-age group and adolescents. A study revealed that children under 12 years old experience more fear and pain than those over 12, in procedures involving needles (26). The lower perception of stress, anxiety, or pain among adolescents observed in the results of this study, specifically in peripheral venipuncture, is consistent with literature that suggests that adolescents have a greater capacity for coping due to their cognitive development and greater control of their emotions when compared to other stages of pediatric age (8).

Considering the complexity of factors that determined the experience of stress, anxiety, or pain, it is worth noting that in this study, nurses identified the administration of oral medication and dressing and undressing among the disturbing procedures, although these do not fall within the scope of what is understood as an invasive procedure.

Pain assessment and management education of health staff, particularly pain management in pediatric age, combined with research assessing professional knowledge, attitudes, and outcomes in pain management, is essential to prevent unnecessary suffering and minimize the negative impact on the quality of life of children exposed to invasive procedures (28) Regarding the nurses that participated in this study, the majority had specific training in pediatric pain management, aligning with the guidelines of the Portuguese Directorate-General of Health (1), which recommends training professionals in pain assessment and management as a best practice criterion for controlling pain during invasive procedures in children. In critical care, health professionals need skills and experience to ensure an environment that is stress-free, safe, and comfortable for a child undergoing an invasive medical procedure (29). The results further suggest that pediatric and child health nursing specialists demonstrated greater awareness of the stress, anxiety, or pain associated with invasive procedures in children and adolescents. In this study, 0.4% of participants reported not using any comfort strategies during invasive procedures. Barriers to pain management in pediatrics among nurses are linked to the biomedical paradigm, which tends to prioritize pharmacological interventions. These often require collaboration with medication prescribed by medical staff and, in their absence, hinder effective action. Additionally, the fear of adverse effects further complicates their use (30). Within this framework, the undervaluation of non-pharmacological strategies also emerges as a barrier.

Specific challenges to implement non-pharmacological strategies may relate not only to the need to develop knowledge but also to professionals' beliefs and attitudes, such as viewing these methods as ineffective or suitable only for mild pain. Moreover, there is a belief that repeated exposure to painful procedures increases pain tolerance (30). The same authors emphasize the importance of reinterpreting pain management routines and fostering knowledge development among nurses. Training interventions targeting knowledge gaps in nurses can positively influence pediatric pain management, enhancing confidence and skills (31). The authors consider that pain management begins with a thorough assessment of the pain history, which is essential for improving the selection and effectiveness of the strategies used.

### Strategies used to promote comfort during invasive procedures

The analysis of the open-ended responses regarding strategies employed to promote comfort during invasive procedures revealed two groups of strategies utilized by nurses: pharmacological and non-pharmacological. Non-pharmacological strategies were further categorized into cognitive-behavioral, sensory, and emotional support techniques. The adoption of non-pharmacological strategies to promote comfort during invasive procedures in pediatric settings is of considerable importance, as evidenced by systematic reviews (26, 32, 33) that include studies from various countries, namely the United States, Turkey, Australia, the United Kingdom, and China.

### Cognitive-behavioral non-pharmacological strategies

According to the participants, preparing the child/adolescent was considered as anticipatory care for the procedure. This preparation was described as tailored to the child's developmental stage, including clear explanations, demonstrations, and interactive feedback. Some authors highlight that for children aged 2–12, preparation and information should be age-appropriate, using storybooks or technological tools, although the authors did not reach conclusive results on their efficacy (32). Nonetheless, other authors suggest that preparation and age-appropriate sharing of procedural information should be prioritized when working with children and adolescents (34). The nurses also recognized the importance of empowering children/adolescents by involving them in their care, offering choices, and helping them understand the situation, as well as involving their parents. Empowerment is considered a critical factor influencing the effectiveness of strategies (32). Distraction techniques mentioned by the participants included storytelling, jokes, humor, using objects, animal interaction, conversations, technology, and unspecified distraction measures. Distraction can be active, involving the child, or passive, where parents or nurses divert the child's attention. While active distraction is more effective, both approaches can significantly contribute to pain management (35).

For children over two years old, various distraction methods have demonstrated some efficacy, such as watching cartoons or movies, listening to stories, interactive computer or video games, using cards, virtual reality, playing with toys, parent- or clown-mediated distraction, squeezing a stress ball, or combining distraction techniques with other cognitive-behavioral strategies like relaxation, guided imagery, hypnosis, or breathing exercises (32). The effectiveness of these strategies varies by age. For example, for children aged 7–12, age-appropriate cartoons, video games, and toys effectively reduce anxiety, fear, or pain during invasive procedures, especially when parents are involved in play (36). From ages 4–11, technology-based distractions like passive virtual reality and video games alleviate pain and fear while increasing self-efficacy in managing pain (35). From ages 6–12, cartoons are more effective than simpler methods like balloon blowing. Interactive and technological tools are highly effective, as are art-based distractions like drawing or coloring, and music (35). Live singing (ages 4–16) is more effective than recorded music, and personal and cultural preferences should guide music selection (37). From ages 8–12, virtual reality proved effective for distraction, while cartoons, television, and video games showed promise from ages 3–12 (37).

The relaxation techniques most frequently mentioned by participant nurses included controlled breathing exercises, blowing bubbles, guided imagery, and stress-ball exercises. Other authors found that breathing exercises effectively reduced pain and anxiety in pediatric burn care, although with limited significance compared to control groups. Adequate training in breathing techniques is recommended before implementing these strategies (38). Blowing bubbles is most suitable for children aged 3–12 but should be avoided in high-infection-risk environments like oncology or burn units. Relaxation techniques effectively reduce pain and fear in adolescents aged 12–18, with breathing exercises showing superior results compared to stress balls (39). Guided imagery has shown promise in reducing pain and heart rate during invasive procedures for children aged 8–12 (40). Nurses also mentioned using positive reinforcement during invasive procedures, consistent with evidence (41). These authors describe verbal and non-verbal reinforcements as effective for pain control and enhancing pediatric care experiences. Nurses must adapt these reinforcements to the child's developmental stage and specific needs.

### Sensory non-pharmacological strategies

Sensory non-pharmacological strategies were also mentioned as among the most used approaches, with sucking being the most frequently referred to, considering both non-nutritive sucking and the sucking of sweetened substances (such as sucrose and breast milk). This was followed by touch-related strategies, including massage, kangaroo care, skin-to-skin contact, positioning, containment, and devices that combined vibration with cryotherapy. Auditory resources, such as music, and environmental management strategies, like pediatric-friendly settings, light and sound control, and minimal handling, were also highlighted.

In preterm newborns, strategies such as non-nutritive sucking, facilitated tucking, and swaddling are considered promising in reducing pain. Non-nutritive sucking reduces pain in term neonates, although there is insufficient evidence supporting the use of these strategies in older children (26). Repeated doses of sucrose solution in very preterm newborns may not be safe, and the appropriate cumulative dose remains unclear in neonates of varying gestational ages (42). Rare adverse effects were reported only with non-nutritive sucking in preterm newborns, including vomiting and reduced oxygen levels (26). In Neonatal Intensive Care Units (NICUs), the combination of sucrose and sucking has shown superiority compared to sucrose alone. However, this superiority is no longer significant when compared to breastfeeding or the administration of breast milk drops using a pacifier, especially when combined with environmental management and/or other sensory/physical strategies like containment (43). In a review of the most effective strategies for managing pain during heel prick procedures in newborns, the use of oral sucrose combined with sucking, administered 2 min before the procedure, was identified as the most effective approach compared to sucrose alone, glucose, expressed breast milk, and non-nutritive sucking (44).

Other interventions, such as white noise, touch/massage, and sensory saturation, were also considered effective but required trained personnel. Strategies like breastfeeding and maternal holding were noted to be challenging to implement consistently due to the difficulty of always ensuring the mother's availability (44). Gaps were identified in the reviewed studies for infants up to three years old, recommended conducting randomized controlled trials (RCTs) on other non-pharmacological interventions, such as touch, environmental management, multisensory bundles, and non-nutritive sucking in infants (26). In the same review, other strategies were identified as potentially effective across all ages up to three years, including the use of toys, massage, touch, applying pressure, sounds, smells, environmental stimulus management, application of heat or cold, vibration, video distraction, and co-bedding. However, these strategies require higher-quality evidence.

The kangaroo care technique was considered effective in relieving pain during invasive procedures in neonates (45) and preterm neonates (46). However, its cultural acceptance must be considered, as studies conducted in China and Iran did not find it to be a beneficial strategy (47). The use of devices combining vibration and cold showed some effectiveness in children aged 6–12 years, although further studies are needed for this age group (35). The authors also warned that this strategy might interfere with analytical results in blood samples, a phenomenon observed in adults. Cold massage proved effective for pain management in children aged 6–12 years (37).

Massage, hugging, and acupressure have shown effectiveness during invasive procedures in neonates, reducing pain, crying duration, oxygen saturation, and heart rate, however, these methods were surpassed in efficacy by breastfeeding (48). The massage is more effective than therapeutic touch in neonates and can be combined with olfactory stimulation and music, forming a multisensory strategy (49). Other sensory non-pharmacological strategies, although not mentioned by the participants, are currently emerging as promising, such as the use of ShotBlocker (50), acupressure, and chewing gum (35).

### Non-pharmacological emotional support strategies

The most frequently cited non-pharmacological emotional support strategies included the presence of parents or significant others, parental collaboration through empowerment in comfort-enhancing strategies, and the use of meaningful objects. The presence of parents is not consensual in scientific literature, with studies supporting and concluding its effectiveness in invasive procedures for controlling pain, fear, and distress, while others indicate negative consequences, such as longer crying duration and increased distress behaviors (33). The presence of the mother during procedures increases the child's pain tolerance, manifested with greater support (37). Despite the evidence based on Bowlby's attachment theory regarding parental involvement in children under three years of age, the parents' presence was found to be not effective in reducing pain in neonates, although it proved effective from 18 months of age on, showing the need to tailor interventions and parental involvement to child development (26). In another review it was evidenced that parental collaboration was effective in pain control in neonates, but it was not limited to distraction techniques alone (47). It also involved sensory strategies mediated by parents, such as the kangaroo method, skin-to-skin contact, as well as nutritive sucking and breastfeeding (47).

A review found significant effects on pain reduction when parents were present during invasive procedures (33). However, the effects on fear, anxiety, and stress varied, with half of the studies concluding that there were no differences in the presence or absence of parents. Some studies found that the presence of anxious parents could increase stress and pain in children, especially between the ages of one and four, where stress behaviors increased further when parents left. Between the ages of four and seven, stress behaviors were negative if anxious parents were present. The authors of the review concluded that having parents present is advantageous because, despite still being controversial, no considerable adverse effects were found, especially in younger children or those with low anxiety levels (33). The presence of parents shows variable results in terms of pain and stress reduction in children, depending on whether the parents are actively involved and feel comfortable, which can lead to positive effects on children, or if parents display overly protective behaviors, which tend to increase pain and stress in children with high anxiety levels (33). The same review adds that further research is needed to identify which types of children and parents benefit most from parental presence, recommending that, in addition to identifying the anxious profile of both the parents and the child (family dynamics), healthcare professionals should allow the parents' presence if both wish, but provide prior training on involvement and adjust their presence and collaboration throughout the procedure.

Most reviews on the effectiveness of non-pharmacological strategies focus on neonates (26) and children up to 12 years old, but there are few reviews specifically addressing adolescents aged 12–18 years (32). This emphasizes the need for various RCT studies on non-pharmacological strategies for children under 12 and reviews for adolescents to enable better evidence-based practices for comfort strategies in pediatric invasive procedures.

Nurses participating in this study reported using a variety of non-pharmacological strategies in pediatrics to promote comfort. Pain and fear management in pediatric invasive procedures is characterized by complexity, inherent in the need to consider not only child development (31) but also the different settings and types of invasive procedures. This complexity increases when associated with previous pain experiences of children/adolescents, cultural context, and individual pain thresholds (31).

The guidelines for the management of acute pain in emergency situations (51) initially recommend non-pharmacological strategies with topical anesthetics, and if these are insufficient, the use of oral analgesia (paracetamol or non-steroidal anti-inflammatory drugs), followed by the association of inhaled anesthetics and, if necessary, stronger drugs such as opioids.

### Pharmacological strategies

The pharmacological strategies mentioned by the participating nurses primarily involved the use of topical anesthetics, followed by analgesia, mild sedation, and other drugs, including inhaled sedatives/analgesics.

Evidence shows that among the various drugs available, topical anesthetics are recommended as the first-line approach because they are considered non-invasive, low-cost, effective, and free of adverse effects (35). The use of nitrous oxide carries some risks and adverse effects, with contraindications. Although it is considered safe and effective for invasive procedures, its routine use is not consensual, and the risks and benefits must be carefully weighed (52). For minor invasive procedures such as venipuncture or intramuscular injections, nitrous oxide can also be effective, but attention should be paid to potential complications. As a gas harmful to the environment, it requires occupational protection, adequate ventilation, and appropriate cleaning systems (53). In pediatric emergency departments, for procedures such as sutures or fracture reductions, other sedative drugs and local anesthetics may be used, but they typically require venous access (53). Currently, intranasal anesthetics are available and can be considered as an alternative (53, 54).

Non-pharmacological strategies, being safe, easy to use, and cost-effective, not only reduce pain and anxiety but also decrease the number of interventions needed to perform procedures and shorten their duration (35). Considering the adverse effects of drugs, non-pharmacological strategies should always be considered. A multimodal approach is recommended for children/adolescents and their families in pediatric nursing practices during invasive procedures. This approach should combine non-pharmacological and pharmacological strategies (35, 48, 55).

### Limitations and future perspectives

Although this study provided important data, these should be interpreted considering some limitations. The perception of nurses, although essential, may not fully reflect the subjective experiences of children. The subjectivity in the perception of pain is a limitation of this study, as different nurses may interpret pain in different ways, thereby influencing the consistency and comparability of the data collected. Future studies could integrate methods such as self-report scales for older children or qualitative reports from parents and caregivers to complement the data. Additionally, it would be useful to explore the impact of specific interventions, such as topical anesthetics or distraction devices, across different age groups. The results provide guidance on invasive and painful procedures and how they can be interpreted by nurses in different age groups. The perception of the severity of pain and distress, and nurses' understanding of the use of pharmacological and non-pharmacological strategies in different age groups can contribute to guidelines and training. A greater understanding among nurses about their practices and interventions could help minimize pain in pediatric settings through evidence-based reflections.

## Conclusion

This study highlights that nurses' perceptions of stress, anxiety, and pain levels caused by invasive procedures in children and adolescents vary by age group, reflecting differences in responses and adaptation to traumatic procedures in pediatrics. Procedures such as lumbar puncture, insertion of probes and catheters, secretion aspiration, and peripheral venous puncture were consistently identified as the most challenging in terms of emotional and physical impact, especially in younger age groups. It was observed that specialized training in child and pediatric health influences the perception of a greater impact of these procedures, emphasizing the importance of a differentiated approach adapted to the pediatric context. The strategies used by nurses to minimize stress, anxiety and pain during invasive procedures predominantly included non-pharmacological approaches, such as cognitive-behavioral, sensory, and emotional support techniques, with less use of pharmacological strategies. An important result of this study was the differences observed in the perception of nurses of the total levels of stress, anxiety, or pain, based on their nursing education: nurses with a degree and nurses with a specialty in child and pediatric health were more aware of childreńs distress levels. These results reinforce the need for ongoing training of healthcare professionals, namely nurses, in specific interventions for stress and pain management in the pediatric context, as well as the adoption of evidence-based strategies to promote the comfort and well-being of children and adolescents undergoing invasive procedures.

## Data Availability

The original contributions presented in the study are included in the article/Supplementary Material, further inquiries can be directed to the corresponding author.
